# Older Adults' Experiences and Expectations of Doctor–Patient Interactions During Early Hospital Care

**DOI:** 10.1111/hex.70207

**Published:** 2025-03-27

**Authors:** George Wells, Kate White, Vasi Naganathan, Natalie Ambrose, Janani Thillainadesan

**Affiliations:** ^1^ Department of Geriatric Medicine Concord Hospital Concord New South Wales Australia; ^2^ Cancer Care Research Unit Susan Wakil School of Nursing and Midwifery, Faculty of Medicine and Health, The University of Sydney Camperdown New South Wales Australia; ^3^ Department of Geriatric Medicine Concord Hospital, Centre for Education and Research on Ageing Concord New South Wales Australia; ^4^ Concord Clinical School, Sydney Medical School, Faculty of Medicine and Health, University of Sydney Camperdown New South Wales Australia

**Keywords:** communication, frailty, hospital, older adult, qualitative

## Abstract

**Background:**

‘People‐centered care’ is one of the World Health Organization's six defining features of quality care and recognizes the importance of tailoring healthcare to individual needs. This is particularly important for older patients who are more vulnerable to complications during their hospitalization. The initial medical assessment in hospital is a vital part of any admission, however, the older patient's experience of this is not well understood.

**Objective:**

The aim of this study was to investigate the perspectives of older patients, exploring their experience and expectations during these critical encounters.

**Methods:**

This was a qualitative study. Semi‐structured interviews were conducted at a major teaching hospital in Sydney, Australia with adult inpatients who were > 75 years old, admitted from the Emergency Department, and had multimorbidity, polypharmacy or frailty. Interviews were transcribed and data were thematically analyzed.

**Results:**

The 20 study participants had a median (range) age of 85 (75–95) years and 13 (65%) were frail. Six themes were identified: (1) addressing the presenting complaint, (2) implicit trust, (3) being understood as an individual, (4) kindness and respect, (5) well‐informed and sometimes shared decision‐making and (6) willingness for challenging conversations.

**Conclusions:**

Our findings highlight that older patients expect holistic and individualized care, extending beyond clinical expertise to encompass key professional and interpersonal characteristics such as effective communication, respect and kindness. The next steps are developing ways to upskill doctors in these aspects and involve older people in the development of training and standards to support the delivery of medical care that aligns with their experiences, expectations and preferences.

**Patient or Public Contribution:**

The study design and interview guide were shaped by feedback from a patient and public involvement (PPI) workshop, which informed the interview process. Study findings were also shared with a PPI panel, whose insights were incorporated into this manuscript. As part of a larger research program, these findings will contribute to the co‐design of educational interventions aimed at improving health professionals' assessment and management of older hospital patients.

## 1 Introduction

Adults aged 65 years or older account for almost half of all hospital admissions [1], and are at a higher risk of hospital‐acquired complications such as delirium, falls and medical complications [2]. There are several guidelines, standards and publications aimed at improving the care of older or frail hospitalized patients. A number are based on the 4Ms framework [3, 4]. The World Health Organization (WHO) also has the Integrated Care for Older People (ICOPE) guidelines [5]. Despite these comprehensive guidelines, there is little qualitative data on patient perspectives regarding their early hospital encounters with doctors. Yet, it is during these early encounters that critical actions need to be initiated to prevent complications, assess frailty and ensure appropriate care pathways including early access to rehabilitation and community support on discharge [3, 5, 6]. Given the medical care team has primary responsibility for implementing these recommendations, understanding the patient's perspective and preferences during early encounters becomes imperative.

Studies have reported on interactions between older patients and doctors in other settings. One study found older patients in primary care settings viewed health problems as important if impacting life values and goals [7]. Others have looked at older patients who present to emergency departments showing patients connected their presenting complaint to their autonomy, expected their complaints to be addressed and also demonstrated a varying degree of preferred involvement in their health care [8, 9]. To our knowledge, there have not been any studies that have focused on patient expectations and experience in the setting of their initial encounters on the ward with their doctors following hospital admission. Systematic reviews have shown a relationship between the patient experience and health outcomes (both self‐rated and objectively measured) across primary and secondary care settings [10]. Some evidence suggests that physicians are not meeting the expectations of patients regarding communication or patient participation during hospital admissions [11]. There is mounting evidence that ‘patient priority centered care’ is associated with reduced health care burden, avoidance of unwanted interventions, and improved outcomes [12, 13, 14]. In primary care settings, evidence suggests older patients with multiple chronic conditions self‐identify achievable goals [15]. Tools have been developed to elicit these goals, such as the My Health Priorities Guide [16] resulting in improved outcomes [17]. This study aims to further our understanding of older patients' initial encounters with doctors in hospital, so that we can identify and address their priorities better.

## 2 Materials and Methods

### 2.1 Setting and Participants

Participants were recruited from medical and surgical wards at Concord Repatriation General Hospital, a university teaching hospital in Sydney, Australia between March 2022 and January 2023. To meet study inclusion criteria, participants had to be ≥ 75 years old, admitted to the hospital wards from the Emergency Department, and have multimorbidity (> 2 medical conditions) and/or polypharmacy (≥ 5 prescription medications) and/or frailty (Clinical Frailty Score > 4). Patients were excluded if they were assessed to lack capacity to consent and/or participate, were too acutely unwell to participate, had documented delirium or reduced level of consciousness at the time of recruitment, had limited proficiency in spoken English, uncorrected significant hearing impairment, were admitted to the intensive care unit (ICU) or were under COVID‐19 precautions.

Recruitment was pragmatically based on the availability of the interviewer (G. W.)—a doctor at the hospital and a study author. On these days, all potential participants were screened for eligibility by the interviewer (G. W.) using an automatically generated list of all inpatients on the hospital electronic medical record admitted within the last 24–72 h. To avoid conflicts of interest and reduce the risk of bias, patients under the care of the investigators were not recruited. Patients identified as eligible for the study were approached and interviewed on the same day as screening. No data were collected for those considered ineligible.

### 2.2 Ethics Statement

All participants were given a participant information sheet and written consent was obtained face‐to‐face. This study was approved by the Sydney Local Health District Human Research Ethics Committee (CH62/6/2022‐001).

### 2.3 Study Design

Drawing on hermeneutic philosophy, this study was guided by an explorative, interpretative qualitative research design [18]. This approach enables researchers to examine the subjective perspective of study participants to inform understanding of the topic. This was undertaken using in‐depth semi‐structured interviews guided by open‐ended questions. The goals were to gather qualitative data in the form of in‐depth insights and rich narrative materials from patients.

### 2.4 Data Sources and Collection

Data were collected through in‐depth, one‐on‐one, face‐to‐face semi‐structured interviews, between a single interviewer (G. W.) and patient participants. Interviews were conducted with the aid of an interview guide (see Appendix S1), which was refined through iterative revisions after the initial interviews. The interview guide included open‐ended questions and then specific prompts based on the 4Ms of geriatric care: what ‘matters most’ (including but not limited to end‐of‐life discussions), medications, mobility and mentation [19]. Interviews were conducted face‐to‐face while participants were inpatients and were audio‐recorded. Interviews were undertaken in the patient's room or in an interview room attached to the ward if there was limited privacy. Recordings were transcribed verbatim by a professional transcription service and verified for accuracy. Interviews were continued until a point of data saturation, where new themes were no longer emerging in simultaneous data analysis and where the sample size was sufficiently diverse and large for exploration of the study aims [20]. Data on participant characteristics were collected from the electronic medical records.

### 2.5 Data Analysis

Data analysis was conducted simultaneously with data collection [21, 22] allowing for the progressive focusing of interviews. Following each interview, a summary of initial reflections was completed by the interviewer. This was accompanied by debriefing between researchers, and the generation of written memos that noted events, emotions and unexpected points to generate an overall impression of the data [23, 24].

Transcripts were analyzed separately and simultaneously by two investigators (G. W. and J. T). Data were analyzed using the principles of thematic analysis as outlined by Braun and Clarke [25]. Transcripts were coded in randomly selected groups of four to five. The study investigators compared codes and discussed emerging concepts. This was repeated until all 20 transcripts were coded at which point early groupings were re‐visited. Through sequential meetings and discussions, themes were coalesced and refined until major themes were determined. Baseline characteristics of participants were reported using descriptive statistics.

### 2.6 Patient or Public Contribution

The study design and interview guide were presented for patient and public involvement at an Early Career Researcher Consumer Engagement Workshop organized by the Sydney Health Partners Geriatric Medicine Clinical Academic Group. Sydney Health Partners is a nationally accredited research translation centre in Australia. Feedback from this workshop shaped the application of the interview topic guide, including supporting the 4Ms framework, refining open‐ended questions and how these were asked by the researcher. Additionally, discussion from this workshop guided practical considerations, such as organizing private spaces for interviews to protect participant privacy in multi‐patient wards.

Study findings were presented to a patient and public involvement panel at the authors' research institution (Centre for Education and Research on Ageing, Department of Geriatric Medicine, Concord Hospital). Their insights emphasized the importance of respectful communication and recognizing the individuality of patients beyond their hospital admission, which informed our interpretation. This study is part of a larger research program, where the findings will inform a co‐design phase aimed at developing educational interventions for health professionals to improve their assessment and management of older patients in hospital.

## 3 Results

### 3.1 Characteristics of Study Participants

During the recruitment period, after initial screening for eligibility criteria using the electronic medical records, 29 inpatients were approached to participate, of whom 20 participated in interviews. Amongst the nine who did not participate, six declined to take part in the study when approached, two were too unwell to participate and one did not have capacity to consent to participate in the study. Of those who declined to participate, reasons included having visitors present at the time of approach, feeling too tired or unwell and, in one instance, dissatisfaction with care. Characteristics of the study participants are summarized in Table 1. The median (range) age of the study population was 85 (75–95) years, and 13 participants (65%) were frail (Clinical Frailty Score > 4).

**Table 1:** Baseline characteristics of study participants (*n* = 20).

Characteristics	Total (*n* = 20)
Female, No. (%)	11 (55%)
Median length of admission (in days) at time of interview (range)	2 (1–3)
Median (range) age in years	85 (75–95)
Country of birth, No. (%)	16 (80%) Australia
	1 (5%) England
	1 (5%) Lebanon
	1 (5%) South Africa
	1 (5%) Italy
Median (range) number of medical conditions	8 (4–19)
Median (range) of medications at admission	9 (3–15)
Clinical Frailty Score	Median (range) 5 (3–6)
	CFS 3: *n* = 4 (20%)
	CFS 4: *n* = 3 (15%)
	CFS 5: *n* = 7 (35%)
	CFS 6: *n* = 6 (30%)
Admitting team, No. (%)	9 (45%) Geriatric Medicine
	5 (25%) Gastroenterology
	3 (15%) Cardiology
	1 (5%) Neurology
	1 (5%) Medical Oncology
	1 (5%) Respiratory

### 3.2 Themes

Thematic analysis of the interview data identified six major themes of the participants' experience and expectations regarding their initial interactions with their doctors.

#### 3.2.1 Theme 1: Addressing the Presenting Complaint

A universal theme was an expectation that the participant's presenting complaint be addressed. In almost all instances, the notion that ‘someone was getting to the bottom of this’ [Participant 19] was viewed as the participant's most important concern.

The presentation to the hospital was seen as due to a specific reason and patients wanted this to be addressed, summarized as ‘a question of getting in, getting treatment and going’ [Participant 11].

‘I'm here asking for your help… I'm the one that's got the complaint, therefore I come to you, and I said, doctor, what can you do to help me?’

[Participant 5]

The expected outcome was to return to the community with a good quality of life. For instance, Participant 4 wanted to get back to her spouse and recognized how important the preservation of her vision was to maintain their lifestyle. Other participants wanted to return to driving, tapestry, charity work and sailing. While functional outcomes were rarely raised by participants as a priority, it was generally agreed to be very important when specifically asked about this.

The participant who felt her presenting complaint had not been fully understood expressed dissatisfaction due to the same: ‘I have mentioned it to people, but nobody's taken much notice’ [Participant 14]. This was in the context of having recently spent time at a residential aged care facility and a desire not to return if she could be cured.

#### 3.2.2 Theme 2: Implicit Trust

Many participants expressed implicit trust in doctors. Often, they came into hospital with the supposition that the doctors ‘are trying to help me. I have to help them help me’ [Participant 6]. Participants expressed a belief that doctors were skilled and perceptive. For instance, in reference to cognitive issues, participants commonly believed that doctors would ‘pick up on it fairly quickly’ [Participant 16] if there were memory problems just through conversation and interactions with their patients.

The demeanor and communication skills of doctors often left patients feeling more confident in their care provision. Many said they ‘felt listened to’ [Participant 19]. Participants spoke of feeling reassured by their interaction and how doctors communicated with them. Doctors were seen as ‘calm’ and ‘to the point’, giving participants a sense that ‘they were going to take care of me’ [Participant 12].

‘You see them cool, calm and collected and making statements that are there and intended to do, inspire you with confidence’.

[Participant 14]

Often participants had had prior interactions with the health care system to inspire their trust. This could be prior personal interactions or experiences with a loved one (e.g., spouses) that inspired trust. Some participants even drew trust from interactions they noticed the doctors having with other patients around them. Participant 9 noted how doctors were ‘just so wonderful’ with a confused patient close by. Another participant had observed that the doctors were diligently checking other patients' social circumstances prior to discharge [Participant 6].

#### 3.2.3 Theme 3: Being Understood as an Individual

Many participants expressed that being understood on a personal level was important; that they were more than just a ‘particular symptomatology’ [Participant 13]. Many participants worried about their hospitalization in the broader context of function and quality of life.

‘Is this going to be my life?… I used to belong to the gym. I do dancing, I never got anyone to do my housework… And suddenly, this whole lifestyle has changed’.

[Participant 17]

Participants expected questions about their home situation. There were some who expected to be asked about their spouse's health, functional status and one participant even felt the quality of the relationship should be explored. Some participants felt it was important for doctors to know something about their personality. Personal stories played a role in how participants perceived themselves and often shaped their wish for independence.

‘I'm a very independent person, you know? I raised my two and I raised my grandchildren, and I've always been chair of a big charity’.

[Participant 16]

#### 3.2.4 Theme 4: Kindness and Respect

The perception that treating teams were caring and empathetic played a large role in how initial meetings with doctors were experienced. It was expected that patients be treated ‘with courtesy and compassion’ [Participant 8] and for the most part this was felt to be the case, with the same participant commenting the team ‘are absolutely fantastic’. Participants noted if doctors were felt to be ‘a bit cold’ [Participant 19].

Participants did not want to feel talked down to: ‘I'm not a doddery 80‐year‐old’ [Participant 16]. They were mostly satisfied with their interactions. In the few instances they felt doctors had been condescending, participants expressed their displeasure:

‘If I was 21 or something, they would listen. …Because you're geriatric, they tend to think you know, you're a bit doolally’.

[Participant 13]

#### 3.2.5 Theme 5: Well Informed and Sometimes Shared Decision‐Making

The degree to which participants wanted to be involved in decisions surrounding their medical care varied. This was a common theme when discussing medications. Many participants wanted to be involved in shared decision‐making with their doctors. These participants were upset if they perceived that they were not involved in decisions, with one participant stating it was disrespectful.

Other participants expected their doctors to adopt a more paternalistic role. They expected that doctors were ‘the specialist in their area’ [Participant 13] and that they knew what was ‘best for me’ [Participant 16]. Others felt this was just the way things were done and ‘whatever you're given you're given’ [Participant 14]. Some participants wanted to be ‘a good patient’ and a few even expressed concern about upsetting the doctors by challenging them or being a ‘nuisance’.

‘I realize that they are the people who are going to recommend what treatment… and I'd only be a nuisance… trying to tell them what to do. No, I'm a good patient’.

[Participant 13]

A common theme was that participants did not mind doctors making the medical decisions so long as these decisions were then clearly explained to the participant verbally, in written summary, or both.

‘The patient doesn't have to know everything, especially in minor details. He wants to be informed’.

[Participant 8]

#### 3.2.6 Theme 6: Willingness for Challenging Conversations

Most participants did not avoid and were accepting of difficult discussions, such as advance care planning, and invariably agreed it was important to be discussed at admission. Some expressed relief at the discussion. Often participants did not have specific ideas about what they would want. In many cases they requested guidance from their treating team or for the treating team to do ‘whatever [they] think is right for me’ [Participant 6].

Many of the participants expressed that they did not wish to be resuscitated. It was rare for participants to be specific. However, the sentiment of ‘I don't want to be kept [alive] unless I'm going to have a reasonable life’ [Participant 9] was a frequent response. Others expressed sentiments of having had a satisfying life and not fearing death. One participant even spoke of regret for previously opting for a pacemaker insertion at 94 years of age [Participant 3]. Some of these participants had already made preparations for their death or had formalized their wishes around advance care plans.

There were a few participants who expressed a wish to remain for full resuscitation. Some expressed this in the context of having nothing to lose so ‘why would I refuse?’ [Participant 6]. Another felt there was an inherent conflict of interest in doctors discussing this as it is their ‘job to help you get better’ [Participant 19]. There was only one participant who found these discussions confronting and appeared to feel that ageism may have affected the decision to make him ‘not for resuscitation’.

‘She said I'm on the do not resuscitate list. That's the first thing she said just about… I wasn't expecting it… But when I sort of realized why, I accepted it because you're a geriatric, you know? So, you've reached the sort of turning off point’.

Participant 20

There was variation on when it was felt this discussion should be had. Some felt that while it was important to discuss, the initial meeting was not the right time as ‘what the patient wants more is words of comfort and reassurance’ [Participant 8]. There was also variation on the expected tone of the discussion. Some wanted discussions to be ‘straight out’ [Participant 2]. Others felt this might ‘frighten [patients] out of their boots’ [Participant 18] and they should be more subtle with a focus on compassion and empathy.

## 4 Discussion

This exploratory qualitative study investigated the experiences of older patients concerning their interactions with doctors in the hospital setting, along with their expectations and preferences during this critical phase of medical care. Through thematic analysis, several overarching themes emerged, including addressing the presenting complaint, implicit trust, individualized understanding, kindness and respect, informed decision‐making and readiness for challenging conversations. Our study sheds light on what older patients value and think are important with regards to their medical care when admitted to the hospital.

‘Addressing the presenting complaint’ was a universal theme for all participants and was generally regarded as the most important aspect of their initial medical consultation. Studies have shown that older patients receiving health care in hospitals are not given opportunities to share their concerns or to tell staff members what was important to them [26]. Though in our study, most participants felt their presenting complaint received adequate attention. An important finding in our study was that while participants only occasionally linked their presenting problem to their functional status, they overwhelmingly identified mobility and function as important issues. This adds justification to the inclusion of ‘mobility’ and ‘matters most’ in the 4Ms‐based guidelines [3, 4, 19].

Despite ‘people‐centered care’ being one of the WHO's six features of quality care there is evidence to suggest that this is often not being met [11, 26]. Older patients as a group vary in their preferences regarding medical decision‐making. Some prefer to take a passive approach expecting the doctors to make the decisions while others prefer a shared decision‐making approach [27, 28, 29]. What was clear in our study was that regardless of the degree of involvement expected by participants in decision‐making, understanding the decisions made was very important to participants. Trust, good communication as well as perceived compassion and kindness influenced participants' relationships with their doctors, a finding echoed in other settings [30, 31]. It is recognized that there is a need to better tailor the training of medical graduates with regards to older patients. Our findings suggest that such training should include discussions about the fact that older people often have implicit trust in their doctors, and the importance of recognizing that this implicit trust influences early interactions with doctors and the degree of expected involvement in decision‐making [32].

End‐of‐life discussions were viewed as important and patients were accepting of these discussions. This suggests doctors should not be apprehensive about having these important discussions with patients. This is in line with other studies in non‐terminally ill older patients [33]. However, whereas most participants were happy to have these discussions, a subset of patients did not feel that the initial meeting with their doctors was the right time to have such discussions. We also found variation in patient wishes surrounding advance care wishes, a finding in keeping with other literature [34]. Abdul‐Razzak and colleagues discussed the importance of assessing a patient's readiness when opening such discussions and also the importance of language used in such discussions [35]. Our findings similarly support literature on ‘breaking bad news’ such as the SPIKES Protocol [36] in assessing patient readiness and accessing appropriate language and settings to engage patients in difficult conversations.

Our study highlights how older patients perceive and wish to be approached with regards to their early encounters with doctors following their admission, and sheds light on how older patients engage with the 4Ms of geriatric care. Each aspect of the assessment was generally seen as important to older patients, however, of equal importance was the way they were explored. It is also clear that physicians need to recognize and understand the diversity in this population and their varying expectations.

Our results should be interpreted in light of the study's limitations. Participants were recruited from a single institution. The homogeneity of participants, both geographic and in terms of hospital setting, may impact whether the themes identified in this study can be generalized to other populations. Further research in other clinical and geographical settings could expand our knowledge of the older person's experience across different healthcare systems. Patients were excluded from this study if they had documented delirium or were assessed to lack the capacity to consent and participate. It should be noted that the experiences of older patients with impaired cognition, particularly severe impairment, are likely to differ from participants in this study and further research is needed into this population group.

## 5 Conclusion

In summary, this study provides in‐depth knowledge of the expectations and experiences of older patients regarding initial interactions with their doctors during hospitalization. Our findings provide important insights that could inform the education and training of doctors on approaching the older hospitalized patient. The healthcare preferences of more diverse populations and those with cognitive impairment issues should be explored.

## Author Contributions

**George Wells:** conceptualization, methodology, writing – review and editing, investigation, formal analysis, project administration, data curation, writing – original draft. **Kate White:** methodology, investigation, formal analysis, writing – review and editing. **Vasi Naganathan:** methodology, investigation, formal analysis, writing – review and editing. **Natalie Ambrose:** methodology, investigation, formal analysis, writing – review and editing, data curation. **Janani Thillainadesan:** conceptualization, methodology, funding acquisition, writing – original draft, supervision, resources, investigation, writing – review and editing, formal analysis, project administration.

## Ethics Statement

This project was reviewed and approved by the Sydney Local Health District Human Research Ethics Committee (CH62/6/2022‐001).

## Consent

All participants were given a participant information sheet and written consent was obtained face‐to‐face.

## Conflicts of Interest

The authors declare no conflicts of interest.

## Supporting information

Supporting information.

## Data Availability

The data that support the findings of this study are available from the corresponding author upon reasonable request.
