# Supplementary material for: Older Adults' Experiences and Expectations of Doctor–Patient Interactions During Early Hospital Care
Source: Health Expect. 2025 Mar 27;28(2):e70207. doi: 10.1111/hex.70207 (PMC11949844; doi:10.1111/hex.70207)
Supplement: Supplementary file 1 — Supporting information. [file HEX-28-e70207-s001.docx]

**Appendix 1: Interview Guide**

*Exploratory questions*

- Tell me about your first experience of meeting the team doctor when you got to the ward?
- What did the doctor ask you about?
- What do you expect the doctor to ask you about when you meet them for the first time on the ward?
- What was the priority for you when the doctor came to see you?
- What do you think the doctor needs to know about you to provide the best medical treatment in hospital?
- What went well during your meeting with the doctor?

*Probes and prompts*

- Was that helpful to you?
- How did you feel when you were asked about that?
- Was that important to you?
- Why is that important to you?
- Did you understand the plan?

*Specific questions based on 4Ms framework*

- When you came to the ward do you recall being asked by the doctors looking after you about memory and thinking/ mobility and function/ medications/ what matters to you/ advance care planning and goals of care?
- If yes, tell us about it, what happened?
- How did you feel about being asked about memory and thinking/ mobility and function/ medications/ what matters to you/ advance care planning and goals of care?
- How would you like the doctor to ask you about memory and thinking/ mobility and function/ medications/ what matters to you/ advance care planning and goals of care?
- Did you expect to be asked about memory and thinking/ mobility and function/ medications/ what matters to you/ advance care planning and goals of care?
- How important is it to you that the doctors looking after you ask you about memory and thinking/ mobility and function/ medications/ what matters to you/ advance care planning and goals of care when you come to hospital as a patient?
